# The chromosome-scale assembly of the willow genome provides insight into Salicaceae genome evolution

**DOI:** 10.1038/s41438-020-0268-6

**Published:** 2020-04-01

**Authors:** Suyun Wei, Yonghua Yang, Tongming Yin

**Affiliations:** 1 Key Laboratory for Tree Breeding and Germplasm Improvement, Southern Modern Forestry Collaborative Innovation Center, College of Forestry, Nanjing Forestry University, Nanjing, 210037 China; 2 College of Information Science and Technology, Nanjing Forestry University, Nanjing, 210037 China; 3 College of Life Sciences, Nanjing University, Nanjing, 210093 China

**Keywords:** DNA sequencing, Next-generation sequencing

## Abstract

*Salix suchowensis* is an early-flowering shrub willow that provides a desirable system for studies on the basic biology of woody plants. The current reference genome of *S. suchowensis* was assembled with 454 sequencing reads. Here, we report a chromosome-scale assembly of *S. suchowensis* generated by combining PacBio sequencing with Hi-C technologies. The obtained genome assemblies covered a total length of 356 Mb. The contig N50 of these assemblies was 263,908 bp, which was ~65-fold higher than that reported previously. The contiguity and completeness of the genome were significantly improved. By applying Hi-C data, 339.67 Mb (95.29%) of the assembled sequences were allocated to the 19 chromosomes of haploid willow. With the chromosome-scale assembly, we revealed a series of major chromosomal fissions and fusions that explain the genome divergence between the sister genera of *Salix* and *Populus*. The more complete and accurate willow reference genome obtained in this study provides a fundamental resource for studying many genetic and genomic characteristics of woody plants.

## Introduction

The Salicaceae family contains the sister genera of *Salix* (willows) and *Populus* (poplars), which are diecious catkin-bearing woody plants. Willows and poplars are distributed widely in many regions of the world. They exhibit high wood production and are commercially important in artificial plantations. Within the Salicaceae family, *Populus trichocarpa* was the first sequenced woody plant^1^, making it a widely adopted model system for various types of genetic studies on tree species. The *P. trichocarpa* v3.0 assembly is ~430 Mb, with a contig N50 and scaffold N50 of 205 kb and 8 Mb, respectively. A total of 41,335 protein-coding genes are predicted in the *P. trichocarpa* genome. In recent years, the genomes of *P. euphratica* Oliv^2^, *P. pruinosa* Schrenk^3^, *P. alba* var. *pyramidalis*^4^, *P. alba*^5^, *P. tremula*, and *P. tremuloides*^6^ have also been sequenced, assembled, and annotated. Moreover, the first draft genome assemblies of a *P. deltoides* (http://phytozome.jgi.doe.gov) and a *P. tremula*×*alba* hybrid (http://aspendb.uga.edu/s717) have been made publicly available online. Compared to poplars, whole-genome-sequencing studies on willow species have been lagging behind; only the genome sequences of *S. purpurea*^7^ and *S. suchowensis*^8^ have been reported. The genome of *S. purpurea* (http://phytozome.jgi.doe.gov) was assembled with Illumina sequencing reads, resulting in a genome assembly of 392 Mb. Within this assembly, 276 Mb (~70%) of sequences were anchored along different willow chromosomes aided by a genetic map^7^. For *S. suchowensis*, a genome assembly covering a total length of 303.8 Mb was obtained from Roche-454 sequencing reads. Anchored with a genetic map constructed for the sequenced individual, 229.2 Mb of sequence assemblies were positioned onto the 19 chromosomes of the *S. suchowensis* genome^8^.

Salicaceae species possess relatively small genomes. However, it is difficult to achieve high-quality genome assemblies for these species using short-read sequencing technologies due to the high heterozygosity and abundant repetitive sequences in their genomes. There is an active demand to improve the currently assembled *S. suchowensis* genome, which shows inaccuracy and low contiguity. In recent years, the single-molecule, real-time (SMRT) PacBio^9^ sequencing and chromosome conformation capture (Hi-C)^10^ techniques have been used to make significant advances in improving the assembly of plant genomes at the chromosomal level. Single-molecule sequencing can generate reads with an average length of >10 kb, which overcomes the restriction of the short reads generated from the Illumina sequencing platform. Additionally, Hi-C technology captures chromatin interactions within the nucleus, which can be used to infer the location of unanchored contigs and, thus, help to reconstruct haplotypes at the chromosomal level. Recently, several high-quality plant genomes have been obtained by using PacBio sequencing and Hi-C technologies^11–13^.

According to the taxonomy of Salicaceae, there are only ~29 species in genus *Populus*^14^. By contrast, over 300 species have been distinguished in genus *Salix*^15^. Poplars generally take the form of large trees, while willows exhibit different growth forms, including large trees, subtrees and small shrubs. Poplars and willows are not only important industrial fiber resources but are also widely used for landscaping. *S. suchowensis* is a small shrub that normally arrives at sexual maturity at one year of age. This species also possesses other characteristics that are favorable for basic genetic studies, such as ease of vegetative propagation and a predisposition to hybridization. Taken together, these characteristics make *S. suchowensis* an ideal model system for revealing the genetic mechanisms underlying the unique biological processes of woody plants. In this study, we aimed to obtain a highly contiguous, chromosome-scale genome assembly by combining PacBio sequencing and Hi-C technologies. The new assembly provides a much better reference genome for *S. suchowensis*, which will facilitate studies on Salicaceae species and serve as a valuable genomic resource for the woody plant research community.

## Results

### Genome sequencing and assembly

In this study, we generated a total of 33.29 Gb PacBio sequencing reads from the SMRT sequencing platform, achieving ~87x coverage of the *S. suchowensis* genome (Suupplementary Table S1). First, the raw PacBio sequencing reads were self-corrected and assembled, which produced assembly groups containing 5346 contigs. Then, 902 redundant contigs (alternative contigs) were identified and removed through sequence alignment analysis. Finally, 3444 primary contigs were retained to reconstruct the haploid pseudomolecule for each chromosome. The obtained genome assembly contains 3444 contigs, covering a total length of 356.5 Mb, with a GC content of 34.94% and a contig N50 of 263.9 Kb (Supplementary Table S2). The 373 largest contigs cover >50% of the genome length, and 1926 large contigs represent ~90% of the genome.

**Fig. 1:**
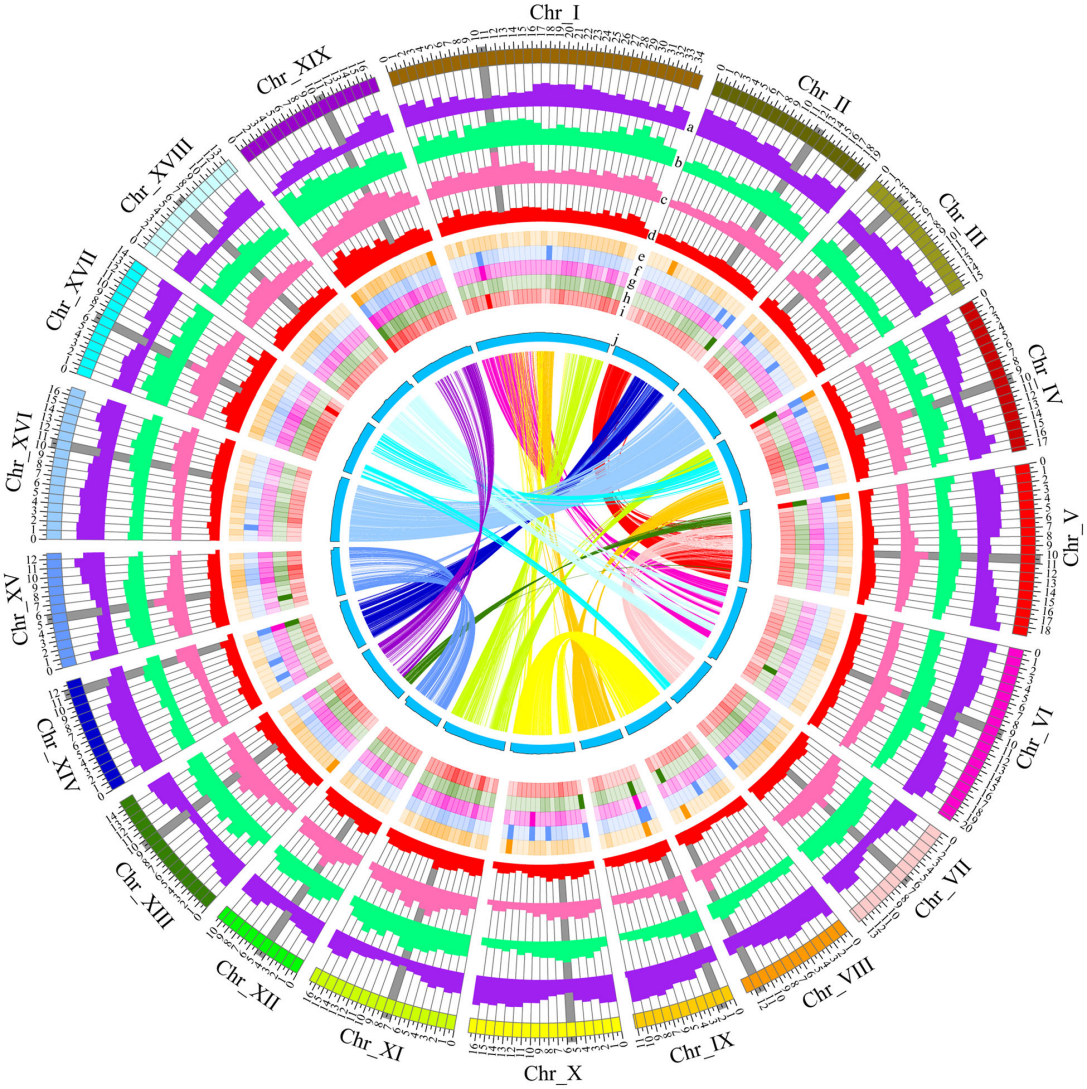
Chromosomal features of *Salix suchowensis*. **a** Gene density, **b** *Copia* density, **c** *Gypsy* density, **d** SSR, **e**–**i** average expression values of genes in five different tissues of willow, and **j** GC content. All of these data are shown in 1 Mb with sliding windows of 200 kb. The inner lines show syntenic blocks on homologous chromosomes. Gray bars indicate the locations of predicted centromeres

**Fig. 2:**
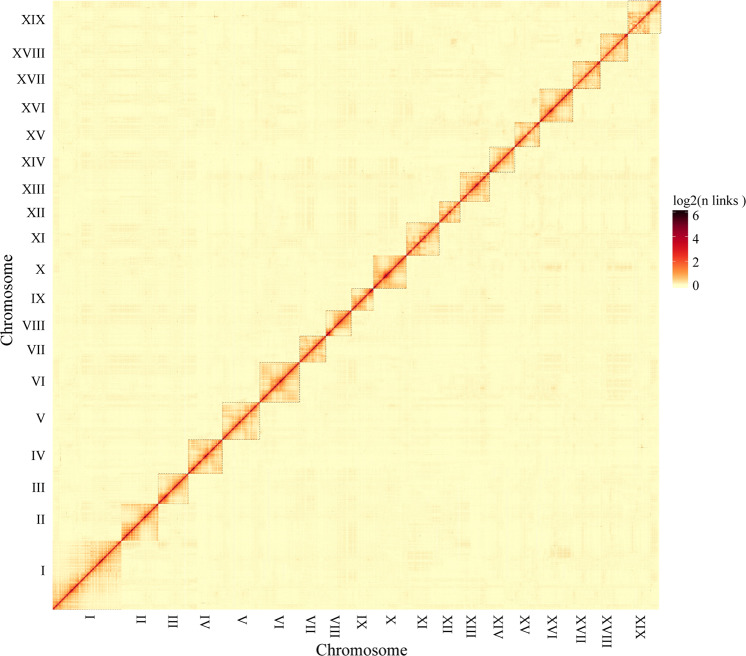
Hi-C contact data mapped on the updated *S. suchowensis* genome v2.0 showing genome-wide all-by-all interactions

In total, 40.48 Gb clean reads were obtained from Hi-C sequencing, covering over 98x of the *S. suchowensis* genome (Supplementary Table S3). We assessed the quality of the Hi-C data, in which 92.56% of Hi-C sequencing reads were mapped to the assembled contigs, 53.60% of which were unique mapped read pairs (Supplementary Table S3). Integrating 81.47% of the valid interaction pairs from the unique paired alignments, the PacBio sequencing assemblies were categorized and ordered to construct chromosome-scale scaffolds, resulting in a total of 1201 contigs, with a scaffold N50 of 16.8 Mb (Table 1 and Fig. 1). Then, the derived contigs were assembled into 19 pseudomolecules by means of agglomerative hierarchical clustering and the use of LACHESIS analysis tools. In total, 339.7 Mb of sequences were anchored along the 19 chromosomes in willow, representing 95.28% of the assembled contigs (Supplementary Table S4 and Fig. 2). Among the anchored contigs (2913), 2262 contigs (309.88 Mb) were oriented on the 19 pseudochromosomes. The longest and shortest pseudomolecules were chromosomes I and XII, with lengths of 34.97 Mb and 10.67 Mb, respectively.

**Table 1:** Statistics for *S. suchowensis* genome v1.0 and v2.0

Category	*S. suchowensis* genome v1.0	*S. suchowensis* genome v2.0
Assembly size (Mb)	303.8	356.5
Total number of contigs	125,988	3444
Contig N50 (bp)	4,094	263,908
Longest contig	110.6 kb	2.19 Mb
Total scaffold number	103,144	1201
Scaffold N50 (bp)	924,964	16,776,717
Longest scaffold (Mb)	6.87	34.98
GC content (%)	34.2	34.9
Number of protein-coding genes	26,599	36,937
Average gene length (bp)	3415	3440
Mean length of exons per gene	5.5	5.6
Repeat content (%)	41.4	47.27

### Genome annotation

We identified a total of 168.5 Mb of non-redundant repetitive elements (47.27% of the genome assembly), 28.27% of which were retrotransposons and 5.32% of which were DNA transposons (Supplementary Table S5), which was higher than the percentage in the previous assembly (125.6 Mb, 41.4%). Similar to *S. suchowensis* genome v1.0, long terminal retrotransposons (LTRs) were found to dominate (21.84%) the repetitive sequences. Among these sequences, *Gypsy* and *Copia* LTRs represented 10.86% and 10.53% of the total genome length, respectively, and accounted for 24 and 23% of the identified repetitive elements, (Fig. 1), whereas non-LTR elements spanned a physical length of 6.67 Mb and accounted for 1.87% of the genome length. In *S. suchowensis* genome v2.0, the class II elements (DNA transposons) represented 5.32% of the genome length. Further analysis revealed that the abundance of repetitive elements in the assembled chromosomes (1432/Mb) was much higher than that in the unanchored scaffolds (378/Mb). By contrast, the content of repetitive sequences was much higher in unanchored scaffolds than in the assembled chromosomes^8^. This comparison indicates that the negative effect of repetitive sequences on genome assembly can be effectively overwhelmed by long sequence reads.

Sequence annotation predicted 36,937 protein-coding genes in *S. suchowensis* genome v2.0 by combining the de novo, homology-based and transcriptome-based predictions (Supplementary Fig. S1). The average gene size was 3440 bp, with 5.6 exons per gene (Table 1). Among the predicted genes, 31,863 were anchored on the 19 chromosomes and covered a total length of 115.10 Mb, with a total exon length of 40.68 Mb (Fig. 1 and Supplementary Table S6). Based on high centromere-associated repeat abundance and a low gene content relative to the other regions of the chromosomes, the centromeres was predicted and positioned in each chromosome (Fig. 1), which revealed four acrocentric chromosomes (Chr_III, Chr_IX, Chr XIV, Chr_XVIII) and 15 submetacentric chromosomes in the genome of *S. suchowensis*. Among the predicted protein-coding genes, 30,260 (81.92%) were supported by transcriptome data from at least one of five tissues, including tender roots, young leaves, vegetative buds, nonlignified stems and bark (Supplementary Table S7). In total, 36,150 (97.87%) predicted genes showed functional annotations in at least one protein database (Supplementary Table S8). Conserved domain features were detected in 40.6% of the protein-coding genes. Additionally, we further annotated the noncoding RNAs and predicted 846 tranfer RNA (tRNA) genes, 261 ribosomal RNA (rRNA) genes, and 172 microRNA (miRNA) genes (Supplementary Table S9).

### Genomic comparison

In this study, we evaluated the genome assemblies of *S. suchowensis* v1.0 and *S. suchowensis* v2.0 (Supplementary Table S10). We sequenced seven randomly selected bacterial artificial chromosomes (BACs) of *S. suchowensis* genomic DNA. Sequence alignment showed that *S. suchowensis* genome v2.0 covered 99.2% of the BAC sequences, while *S. suchowensis* genome v1.0 covered 93.1% of the BAC sequences (Supplementary Table S11). The BAC sequence alignment also showed that v2.0 exhibited better continuity and higher precision than v1.0. We further evaluated the quality of the new assembly with ~30 Gb of Illumina paired-end reads. The mapping ratio for the Illumina reads was 78.11% for v1.0, which increased to 90.38% for v2.0 (Supplementary Table S12). To assess the gene coverage, a total of 85,962 unigenes derived from RNA-Seq data for *S. suchowensis*^8^ were mapped to the two versions of the genome assemblies. More than 99.4% of these unigenes could be mapped to v2.0 (Supplementary Table S13), while ~97.6% of them could be mapped to v1.0. The completeness of the predicted genes in v2.0 was also assessed by sequence alignment with 1440 conserved Embryophyta genes from Benchmarking Universal Single-copy Orthologs (BUSCO)^16^, and the results showed that coverage of these genes was 94.8% and 89.6% for v2.0 and v1.0, respectively (Supplementary Table S10).

A total of 30,265 genes in *S. suchowensis* v2.0 were divided into 21,107 gene clusters, among which 18,692 gene clusters (23,397 genes) were shared with *S. suchowensis* v1.0, and 20,086 gene clusters (28,127 genes) were shared with *P. trichocarpa* v3.0 (Supplementary Fig. S2). The comparison of the protein-coding genes in *S. suchowensis* genome v1.0 with those in *S. suchowensis* genome v2.0, revealed that 25,891 genes in v1.0 were highly homologous to 31,694 genes in v2.0, indicating that the annotated genes in the updated genome were more complete than those in the previous version and included more multicopy genes. Furthermore, 5243 version-specific genes in v2.0 were analyzed for their distribution on the 19 chromosomes and among the unclustered contigs. Among these genes, 1743 were distributed among unclustered contigs, and the rest were distributed on the assembled chromosomes (Supplementary Table S14). GO enrichment analysis showed that the functions of these version-specific genes mainly included the respiratory electron transport chain, NADH dehydrogenase activity, ATP synthesis-coupled electron transport, and oxidative binding (Supplementary Fig. S3). The better gene coverage of the updated genome will greatly facilitate functional genomics studies in the future.

### Gene family analysis

**Fig. 3:**
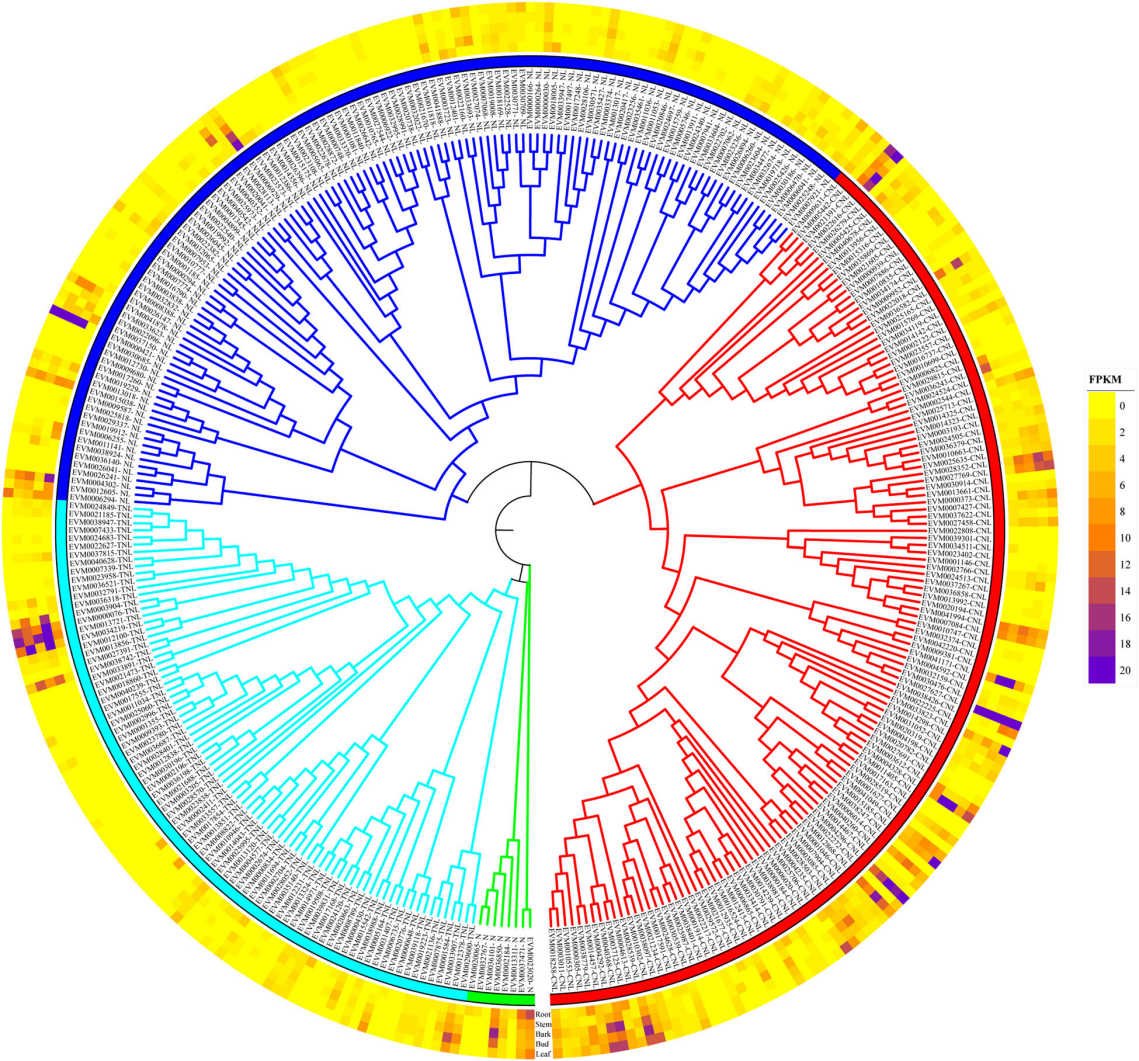
Phylogenetic tree of the nucleotide-binding site (NBS) domain *R* gene family in *S. suchowensis* genome v2.0. Each protein is encoded with the gene number and domains present. Different colors correspond to different subfamilies. Distinct expression profiles of gene members based on RNA-Seq revealed different expression patterns among five tissues, including tender roots, young leaves, vegetative buds, nonlignified stems, and bark^8^

We characterized some important gene families in *S. suchowensis* genome v2.0. *R* genes containing nucleotide-binding site (NBS) domains play a vital role in conferring resistance to plant disease. In *P. trichocarpa*, 402*R* genes containing an NBS domain were detected^17^, including 143 *coiled-coil-NBS-LRR* (*CNL*) genes, 93 *TIR-NBS-LRR* (*TNL*) genes, 115 *NBS-LRR* (*NL*) genes, and 51 *NBS* genes, whereas 346*R* genes containing an NBS domain were identified in *S. suchowensis* v2.0, including 132 *CNL* genes, 83 *TNL* genes, 123 *NL* genes, and eight *NBS* genes (Fig. 3), suggesting either faster shrinkage or slower gain of *R* genes containing an NBS domain in *S. suchowensis* than in *P. trichocarpa*, especially for the *NBS* genes (51 vs. 8). In *P. trichocarpa*, chromosome XIX contains >70*R* genes, most of which are clustered at the peritelomeric end of this chromosome^18^. In *S. suchowensis* v2.0, chromosome XIX contains 107*R* genes, most of which are also located at the peritelomeric end.

**Fig. 4:**
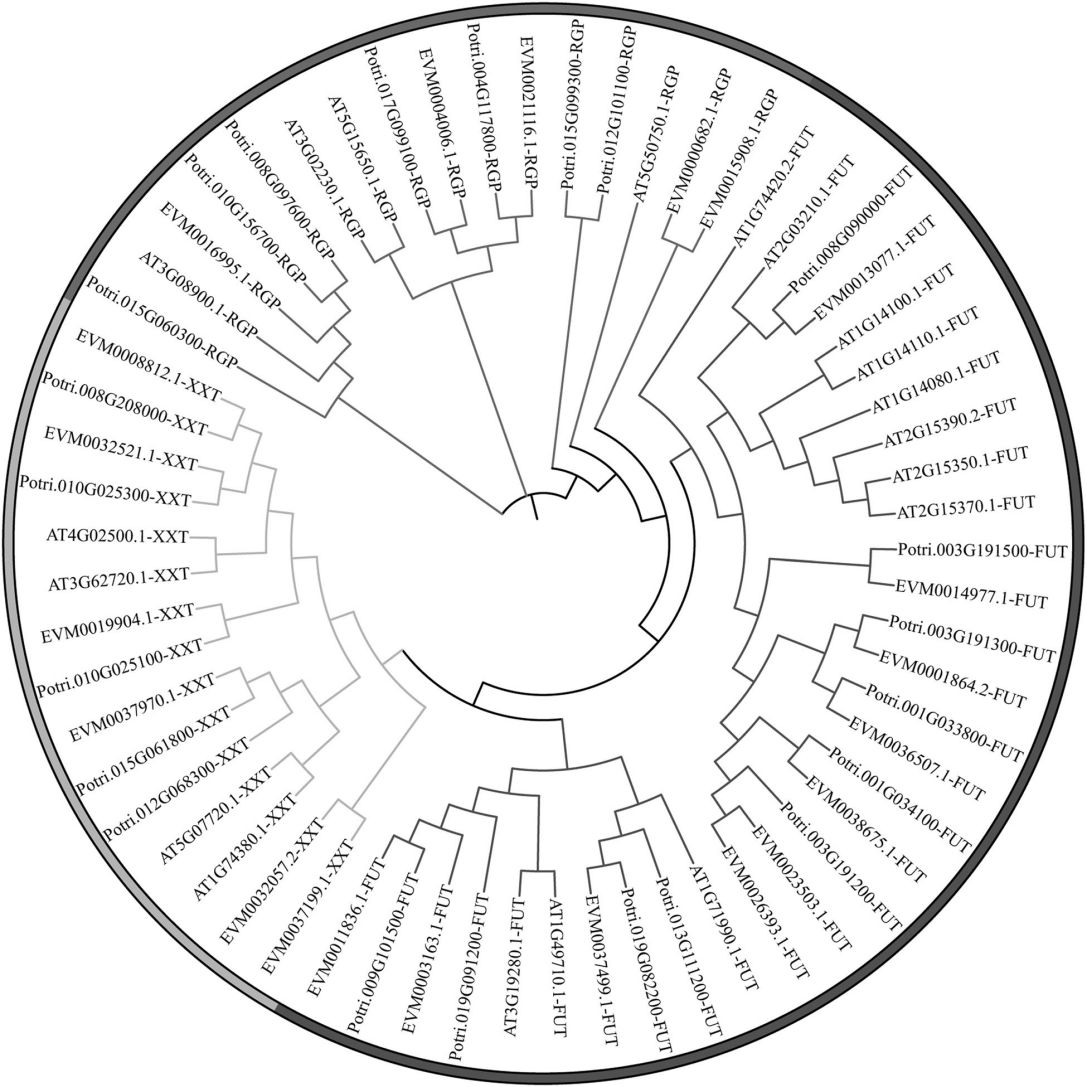
Phylogenetic analysis of genes involved in cellulose and hemicellulose biosynthesis in *Salix*, *Populus*, and *Arabidopsis*. Gene names starting with “Potri” are genes of *P. trichocarpa*, “EVM” indicates genes of *S. suchowensis*, and those starting with “AT” are genes of *A. thaliana*

Wood formation is a unique biological process that occurs in woody plants. In this study, we dedicated special efforts to the analysis of genes involved in wood formation. Wood mainly consists of cellulose, hemicellulose and lignin. We annotated 28 gene families involved in cell wall formation, which included 296 genes involved in cellulose and hemicellulose biosynthesis, including ten fucosyltransferase (FUT) genes, six xyloglucan xylosyltransferase (XXT) genes, five hydroxyproline-rich glycoprotease (RGP) genes, 275 glycoside hydrolase (GH) genes, and 75 genes involved in lignin biosynthesis in *S. suchowensis* v2.0 (Supplementary Table S15). Phylogenetic analysis showed that the numbers of FUT, XXT, and RGP orthologous genes are similar in *S. suchowensis*, *P. trichocarpa*, and *Arabidopsis thaliana* (Fig. 4), but the number of GH orthologous genes in *Arabidopsis* is significantly lower than that in Salicaceae (Supplementary Fig. S4), especially for the GH17, GH18, and GH31 subfamilies. We propose that these gene subfamilies might play a role in secondary cell wall formation, which is a process unique to woody plants. Lignin is an amorphous polymer related to cellulose that provides rigidity and, together with cellulose, forms the woody cell walls of plants. In *S. suchowensis* v2.0, the 75 genes involved in lignin biosynthesis were clustered in ten gene families (Supplementary Fig. S5). In general, willow contains more genes involved in lignin biosynthesis than *Arabidopsis* (75 vs. 34) and fewer genes involved in lignin biosynthesis than *Populus* (75 vs. 90). In detail, the genes coding p-coumarate-3-hydroxylase (C3H), trans-cinnamate 4-hydroxylase (C4H), caffeic acid O-methyltransferase (COMT), ferulate-5-hydroxylase (F5H), and hydroxycinnamoyl-CoA shikimate/quinate hydroxycinnamoyl transferase (HCT) occur in multiple copies in *S. suchowensis* and *P. trichocarpa*, whereas they occur in a single-copy in *A. thaliana*. In particular, COMT, a potential target enzyme for modifying the composition of lignin in plants, is a single-copy gene in *A. thaliana*, while there are nine and 13 copies of *COMT* in *S. suchowensis* and *P. trichocarpa*, respectively (Supplementary Table S15).

Willows are characterized by an unusually high content of salicylic acid in their secondary metabolites^19^. Salicylic acid is a phytohormone that regulates signal transduction pathways involved in the defense against biotic and abiotic stresses. Gene annotation showed that there were 827 genes involved in salicylic acid biosynthesis and metabolism in *S. suchowensis* genome v2.0. The KEGG pathway analysis indicated that these genes were enriched in plant immune signaling to induce systemic acquired resistance, such as plant hormone signal transduction, MAPK signaling pathway-plant, plant-pathogen interaction, and alpha-linolenic acid metabolism pathways (Supplementary Fig. S6). The number of genes related to salicylic acid biosynthesis and metabolism is much higher in *Populus* than in *Arabidopsis* (1397 vs. 125, respectively), suggesting that salicylic acid may be involved in signal transduction processes specific to trees, such as plant hormone signal transduction and wood formation.

### Whole-genome duplication analysis

**Fig. 5:**
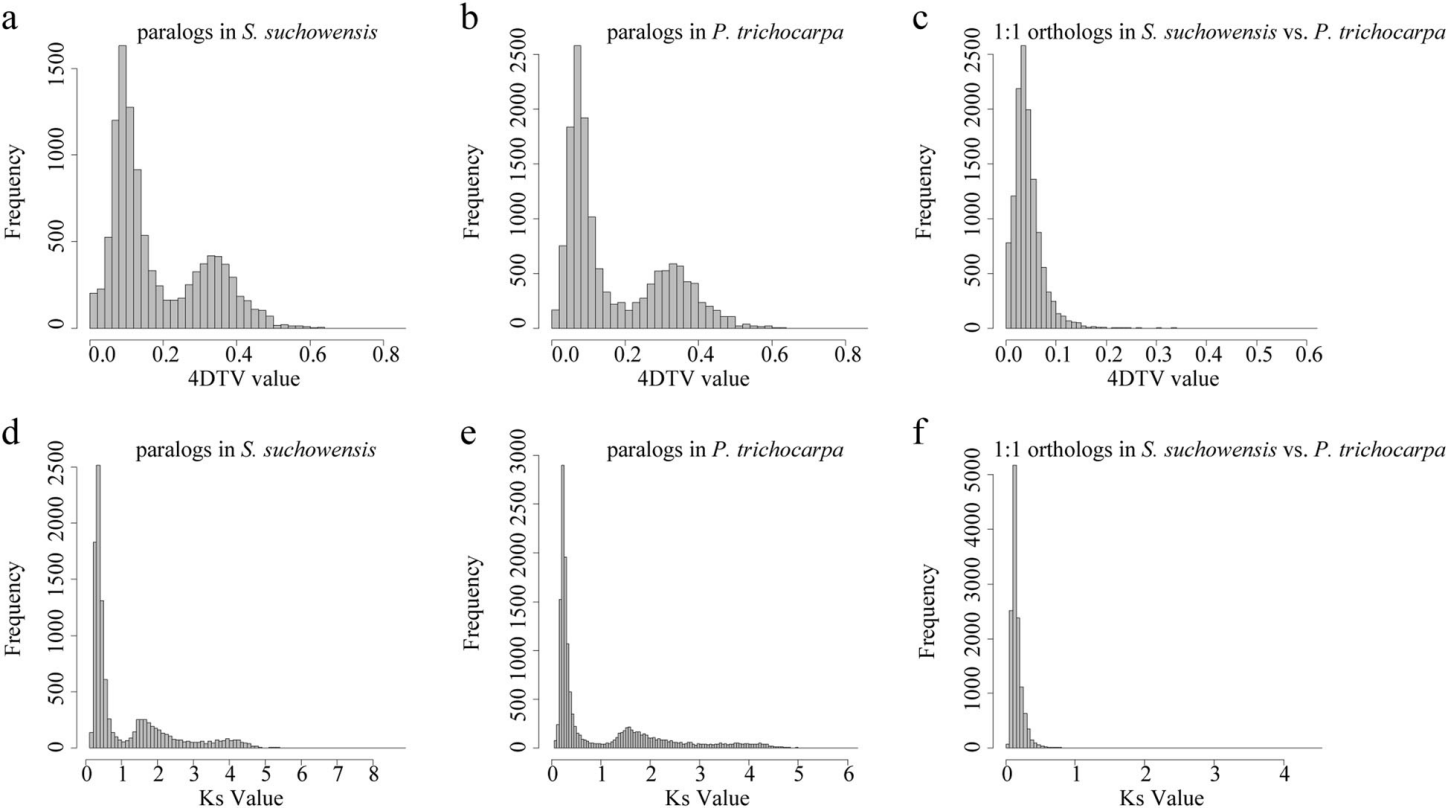
Four-fold synonymous third-codon transversion (4DTV) and the number of synonymous substitutions per synonymous site (Ks) for each homologous gene pair in *S. suchowensis* genome v2.0 and *P. trichocarpa* genome v3.0. **a** 4DTV values for paralogs in *S. suchowensis* v2.0, **b** 4DTV values for paralogs in *P. trichocarpa* v3.0, **c** 4DTV values for single-copy orthologs between *S. suchowensis* v2.0 and *P. trichocarpa* v3.0, **d** Ks values for paralogs in *S. suchowensis* v2.0, **e** Ks values for paralogs in *P. trichocarpa* v3.0, **f** Ks values for single-copy orthologs between *S. suchowensis* v2.0 and *P. trichocarpa* v3.0

**Fig. 6:**
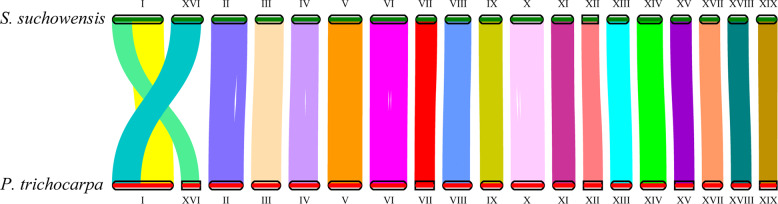
Syntenic comparison of the homologous chromosomes between *S. suchowensis* genome v2.0 and *P. trichocarpa* genome v3.0

We detected 11,037 and 15,092 paralogous gene pairs within the genomes of *S. suchowensis* and *P. trichocarpa*, respectively. We also identified 12,710 single-copy orthologous gene pairs between these two lineages. We calculated the fourfold synonymous third-codon transversion (4DTV) value for each gene pair. Plotting the 4DTV values for the paralogous gene pairs revealed two peaks in both willow and poplar (Fig. 5a, b). The peak associated with salicoid duplication appeared at 4DTV values of 0.101 and 0.082 in *S. suchowensis* and *P. trichocarpa*, respectively. Plotting the 4DTV values for single-copy orthologous gene pairs revealed a peak at a 4DTV value of 0.04 that was associated with the divergence of these two lineages (Fig. 5c). The comparison of the peak positions showed that the divergence of poplar and willow occurred after salicoid duplication in each lineage. The conclusion was further confirmed by analyzing the number of synonymous substitutions per synonymous site (Ks) for each gene pair. Salicoid duplication occurred at Ks values of 0.365 in *S. suchowensis* and 0.283 in *P. trichocarpa*, and the divergence of these two lineages occurred at a Ks value of 0.158 (Fig. 5d–f). Notably, the peaks associated with salicoid duplication were found to not overlap in these two lineages. The same scenario was also observed in previous reports^20^. The analysis of nucleotide substitution rates revealed that this characteristic was caused by the molecular clock clicking more slowly in willow than in poplar^8^. The visualization of synteny blocks (Fig. 6) revealed high collinearity in *S. suchowensis* and *P. trichocarpa* for 17 chromosomes. As found in previous studies^21–23^, large interchromosomal rearrangements were detected between chromosome I and chromosome XVI, suggesting that major chromosomal fusions and fissions occurred after salicoid duplication, leading to the divergence of the two sister genera.

**Fig. 7:**
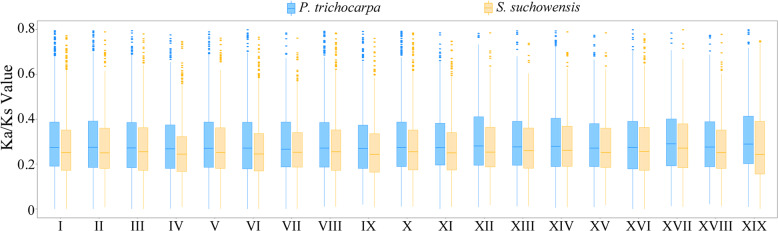
Boxplot of Ka/Ks values for the paralogous genes that arose from salicoid duplication on each chromosome in *S. suchowensis* and *P. trichocarpa*

Furthermore, we examined the extent of sequence divergence by estimating the number of nonsynonymous substitutions per nonsynonymous site (Ka)/Ks for paralogous genes arising from salicoid duplication in both *S. suchowensis* and *P. trichocarpa* (Fig. 7). The average Ka/Ks value was 0.286 for *S. suchowensis* and 0.311 for *P. trichocarpa*, suggesting that salicoid duplicated genes are subject to stronger purifying selection in *S. suchowensis* than in *P. trichocarpa* (Supplementary Table S16). At the chromosome level, the average Ka/Ks values for the salicoid duplicated genes ranged from 0.30 to 0.34 for the chromosomes of *P. trichocarpa*, whereas the corresponding values ranged from 0.27 to 0.3 in *S. suchowensis* (Supplementary Table S16). Thus, stronger purifying selection was observed for each chromosome in *S. suchowensis* than in *P. trichocarpa*, which would lead to the faster loss of duplicated genes in willow than in poplar and ultimately result in a smaller gene content in *S. suchowensis* than in *P. trichocarpa*^20^.

## Discussion

We proposed an improved chromosome-scale assembly of the *S. suchowensis* genome using PacBio long reads and Hi-C technology. The emergence of PacBio sequencing has led to a significant increase in contig N50 sizes compared to previous short-read assemblies. The first assembly of the *S. suchowensis* genome based on Sanger sequencing presents limitations due to its fragmentation and low continuity (Table 1). By combining single-molecule sequencing and Hi-C technology, *S. suchowensis* genome v2.0 spanning 356.5 Mb was obtained, and the contig and scaffold N50s are 264 Kb and 16.8 Mb, respectively, indicating high contiguity of the assembly. Despite the high heterozygosity of the *S. suchowensis* genome, we used Hi-C technology to accurately assign the final assembled contigs to the chromosomal positions and constructed 19 chromosomes of the *S. suchowensis* genome, accounting for 95.29% of the assembly. We accurately compared the quality of *S. suchowensis* genome v2.0 with that of the previous short-read assembly from a variety of aspects, verifying that our updated assembly achieved higher contiguity and quality in its extensive coverage of the genome and higher mapping ratios of the genomic reads, RNA-Seq short reads and conserved genes, suggesting that our assembly is a good alternative resource for diploid willow.

Compared with poplar genomes, willow genomes are generally smaller and contain fewer protein-coding genes. Using k-mer analysis, it was estimated that the genome size of *S. suchowensis* was ~425 Mb^8^, while that of *S. purpurea* was ~450 Mb^7^, both of which are slightly smaller than the estimated 485 Mb *P. trichocarpa* genome^1^. The assembled genome size of *S. purpurea* v1.0 is ~392 Mb, with 37,865 putative protein-coding genes^7^, while the sizes of the recently released *S. purpurea* genome v5.1 and v3.1 are ~329 Mb and ~313 Mb, with predicted coding genes of 35,125 and 34,464, respectively. The published genome size of *S. suchowensis* v1.0 is ~304 Mb, with only 24,931 putative protein-coding genes on 19 chromosomes^24^. Using PacBio sequencing long reads, the size of our improved genome assembly for *S. suchowensis* was 356.5 Mb, and 36,937 putative protein-encoding genes were predicted in the whole genome. With the obvious improvement in the contiguity of *S. suchowensis* genome v2.0, a greater number of genes were annotated than in the previous assembly. However, only 31,863 anchored and oriented genes were found on 19 chromosomes of the *S. suchowensis* genome, and the average Ka/Ks ratios of salicoid duplicated gene pairs on each chromosome in *S. suchowensis* were smaller than those in *P. trichocarpa*, suggesting that willow might be subjected to stronger purifying selection, which is consistent with previous studies indicating that willows have evolved faster than poplars, resulting in a reduced number of predicted genes and an overall smaller genome size^8,20^.

In summary, the development of genomic assembly technologies makes it possible to produce chromosome-level scaffolds in a rapid and cost-effective way. The updated *S. suchowensis* genome v2.0 includes a high-quality chromosomal-level assembly and many important genes, offering novel insights into genome evolution, functional innovation and key regulatory pathways in *Salix* and providing excellent genetic resources for comparative genome studies among Salicaceae species.

## Materials and methods

### Genome sequencing

The same material of female cultivated shrub willow used for the first assembly was selected from the germplasm resources of *S. suchowensis* preserved at Nanjing Forestry University. Fresh young leaves were collected and immediately frozen in liquid nitrogen for genomic DNA isolation and library construction. According to the recommendations of Pacific Biosciences, genomic DNA was fragmented to a target size of ~20 kb to construct the PacBio genome sequencing library. All the DNA libraries were sequenced using the PacBio platform (Pacific Biosciences, CA, USA) with P6-C4 sequencing chemistry. The genomic DNA was sheared to construct short insertion paired-end (PE) libraries following the manufacturer’s instructions (Illumina, CA, USA). All libraries were sequenced at 2×100 bp on the Illumina HiSeq X ten platform. All sequencing procedures were performed by the BioMarker Technologies Company (Beijing, China).

### Genome assembly

The PacBio SMRT-Analysis package (https://www.pacb.com) was used for the quality control of the raw polymerase reads; sequencing adaptors were removed, and low-quality short reads were filtered. Owing to the high error rate of the raw PacBio long reads, we first used the error correction module embedded in Canu^25^ to correct these errors. Then, the high-quality PacBio clean reads were de novo assembled by using Canu with default parameters, which produced assembly agglomerations containing 5346 contigs for the preliminary assembly. Owing to the high heterozygosity of the *S. suchowensis* genome, the clean PacBio subreads were aligned back to the assembled contigs by using BLASR^26^, and the contigs were further corrected with Quiver from the SMRT-Analysis package. The Illumina short reads obtained from the *S. suchowensis* genome were aligned onto the optimized contigs using BWA-MEM^27^, and parsing was performed with SAMtools^28^. As a result, 3444 primary contigs were retained to reconstruct the haploid pseudomolecule for each chromosome.

### Chromosome-scale assembly with Hi-C data

Hi-C libraries were constructed from fresh leaf tissue of *S. suchowensis*, which is the same line that we sequenced for genome assembly. The Hi-C libraries were sequenced on the Illumina HiSeq X ten platform (Illumina, CA, USA) at the BioMarker Technologies Company (Beijing, China). Owing to the limitation of the short reads involved in the Hi-C procedure, we used HiC-Pro^29^ to process these Hi-C sequencing data, including the mapping of the Hi-C sequencing reads to the assembled contigs with the BWA-aln aligning algorithm without any mismatches^30^ and with the detection of valid contacts. The preassembled contigs split into segments of 50 kb on average, combined with uniquely matched Hi-C data, were clustered, ordered and directed onto the pseudochromosomes with LACHESIS software^31^. To improve the chromosome-scale assembly quality, manual adjustment of orientation errors with obvious discrete chromatin interaction patterns was performed. The final chromosome assemblies were divided into 100 kb bins with equal lengths, and the interaction signals generated by the valid mapped read pairs between each bin were visualized in a heat map.

### Genome annotation

We used a combination of de novo prediction and homology-based searches to annotate transposable elements in the genome. To construct a de novo repeat library, the specific transposable elements in our assembly were first screened using LTR FINDER^32^, RepeatScout^33^ and PILER-DF^34^, and these repetitive sequences were then classified into families with PASTEClassifier^35^. Finally, RepeatMasker^36^ was applied to perform a homology-based repeat search using both the de novo repeat library and the known Repbase^37^ transposable elements database.

The repeat-masked genome assembly was used for annotating gene models through a combination of ab initio prediction, homology-based prediction and RNA-Seq-assisted prediction methods. Five different ab initio gene prediction programs were used to identify gene models. In the homology-based prediction, protein sequences from *Arabidopsis thaliana*, *Oryza sativa*, *Populus trichocarpa*, and *Eucalyptus grandis* downloaded from Phytozome (https://phytozome.jgi.doe.gov) were aligned to the assembly with TBLASTN^38^, and then GeMoMa^39^ was used to construct exact gene models from all initially aligned coding sequences. Additionally, RNA-Seq data from five different tissues of *S. suchowensis*, including the roots, stems, leaves, buds and bark, were mapped to the genome assembly using HISAT and Stringtie^40^. TransDecoder (http://transdecoder.github.io) and GeneMarkS-T^41^ were used to identify transcripts from the mapping results. Finally, all gene models predicted from the above three approaches were integrated by using EvidenceModeler (EVM) v1.1.1^42^ to generate consensus gene sets and were filtered with PASA^43^ to detect spliced gene structures and generate non-redundant gene models.

Functional annotations of the predicted genes were further assigned with BLAST^44^ according to the best match of the alignments against the non-redundant plant protein database and non-redundant nucleotide and protein sequences from the NCBI^45^. The Gene Ontology (GO) terms for each gene were obtained from the GO database^46^. The protein motifs and domains were predicted by using InterProScan^47^ to search against various domain databases. The tRNA genes were searched by using tRNAscan-SE^48^ software with the eukaryote parameters. Additionally, microRNA and rRNA genes were identified by using INFERNAL^49^ to search the miRBase^50^ and Pfam^51^ databases, respectively.

### Assessment of genome assembly and annotation

The *S. suchowensis* BAC sequences were aligned to the updated *S. suchowensis* genome using the MUMmer^52^ program. We mapped DNA sequencing data (>50×) from the Illumina HiSeq X ten platform against the first assembly and the updated assembly using BWA-MEM software^27^ and then compared the mapping status of the two genomes with SAMtools^28^. The transcriptomic short reads derived from different tissues of *S. suchowensis* (including the roots, stems, leaves, buds, and bark) from the previously published genome^8^ were aligned to both genome assemblies with STAR^53^. BUSCO^16^ was used to evaluate the completeness of the assembled genome. Orthofinder^54^ was used to classify the orthologs between *S. suchowensis* v2.0 and *P. trichocarpa* v3.0. We used BLASTP^44^ to identify the common genes between *S. suchowensis* v2.0 and *S. suchowensis* v1.0. GO enrichment analysis was carried out with the clusterProfiler^55^ R package.

### Gene family analysis

We used two software programs, BLASTP^44^ and HMMER^56^, to search for homologous proteins harboring the conserved domains of related gene families in *S. suchowensis* genome v2.0 with an expected value (*e*-value) threshold of <1e-10. The predicted protein sequences were further confirmed using both the NCBI conserved domain database (CDD)^45^ tool and the Simple Modular Architecture Research Tool (SMART)^57^ to analyze the conserved domains. The final deduced full-length amino acid sequences were aligned by using the ClustalW program with default parameters^58^. The phylogenetic tree was constructed using MEGA X via the neighbor-joining (NJ) method with 1000 bootstrap iterations^59^ and visualized using the Interactive Tree of Life (iTOL) web tool (http://itol.embl.de)^60^.

We performed a pathway enrichment analysis at the KEGG pathway database (http://www.genome.jp/kegg) to identify enriched metabolic or signal transduction pathways associated with salicylic acid. The classification of biological terms and the analysis of the enrichment of the salicylic acid biosynthesis and metabolism clusters in the KEGG pathways were completed with the clusterProfiler R package^55^.

### WGD analysis

We identified the collinearity within the assembled *S. suchowensis* v2.0 chromosomes, as well as that between the *S. suchowensis* v2.0 and *P. trichocarpa* v3.0 chromosomes. By running a BLASTP^44^ alignment for each genome pair, we searched for putative paralogous and orthologous genes within and between genomes. Fourfold degenerate sites were located, and the 4DTV values were calculated using in-house Perl scripts. The 4DTV range associated with salicoid duplication was determined by plotting the distribution frequency histogram of 4DTV values. For each salicoid duplicated gene pair, Ka and Ks values and Ka/Ks ratios were calculated using the Nei-Gojobori algorithm implemented in KaKs_Calculator 2.0^61^. The boxplot of Ka/Ks values was generated with R software (version 3.6.1; www.r-project.org). The synteny blocks were identified and visualized by using the MCSCAN toolkit implemented in Python [https://github.com/tanghaibao/jcvi/wiki/MCscan-(Python-version)]. The image of the entire *S. suchowensis* genome was produced using Circos^62^.

## Supplementary information

Supplementary Figures

Supplementary Tables
