# Supplementary material for: The chromosome-scale assembly of the willow genome provides insight into Salicaceae genome evolution
Source: Hortic Res. 2020 Apr 1;7:45. doi: 10.1038/s41438-020-0268-6 (PMC7109076; doi:10.1038/s41438-020-0268-6)
Supplement: Supplementary file 1 — Supplementary Figures [file 41438_2020_268_MOESM1_ESM.doc]

**Supplementary Figures**


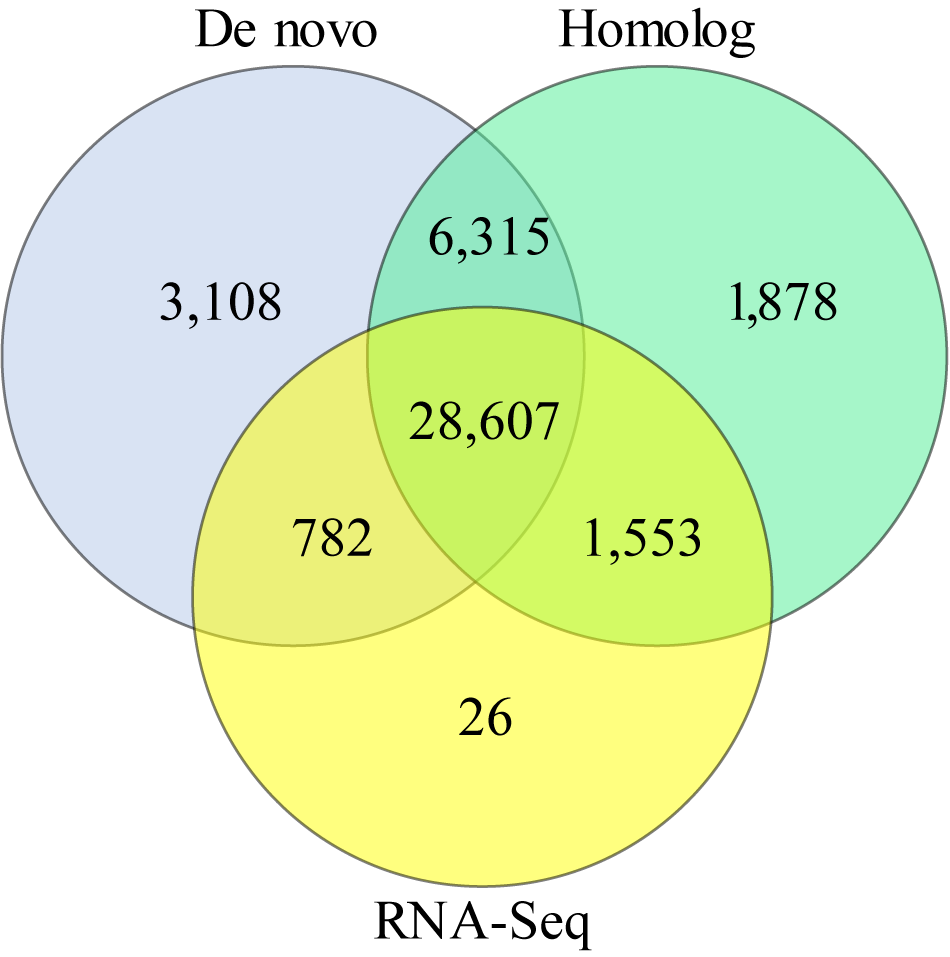


**Fig. S1** Distribution map of integrated gene number derived from three prediction methods


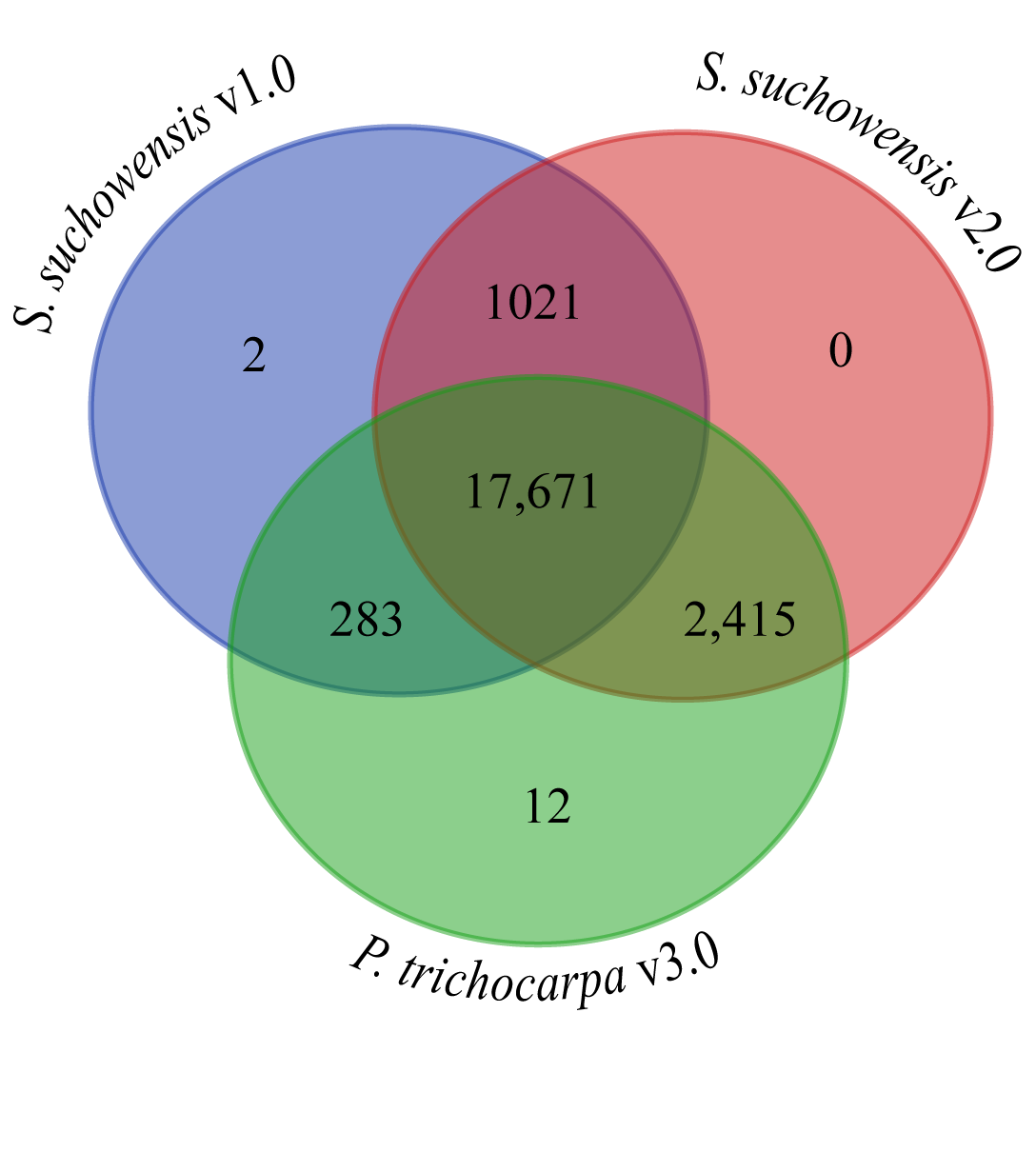


**Fig. S2** Venn diagram about the gene clusters shared by *S. suchowensis* genome v2.0, *S. suchowensis* genome v1.0, and *P. trichocarpa* genome v3.0


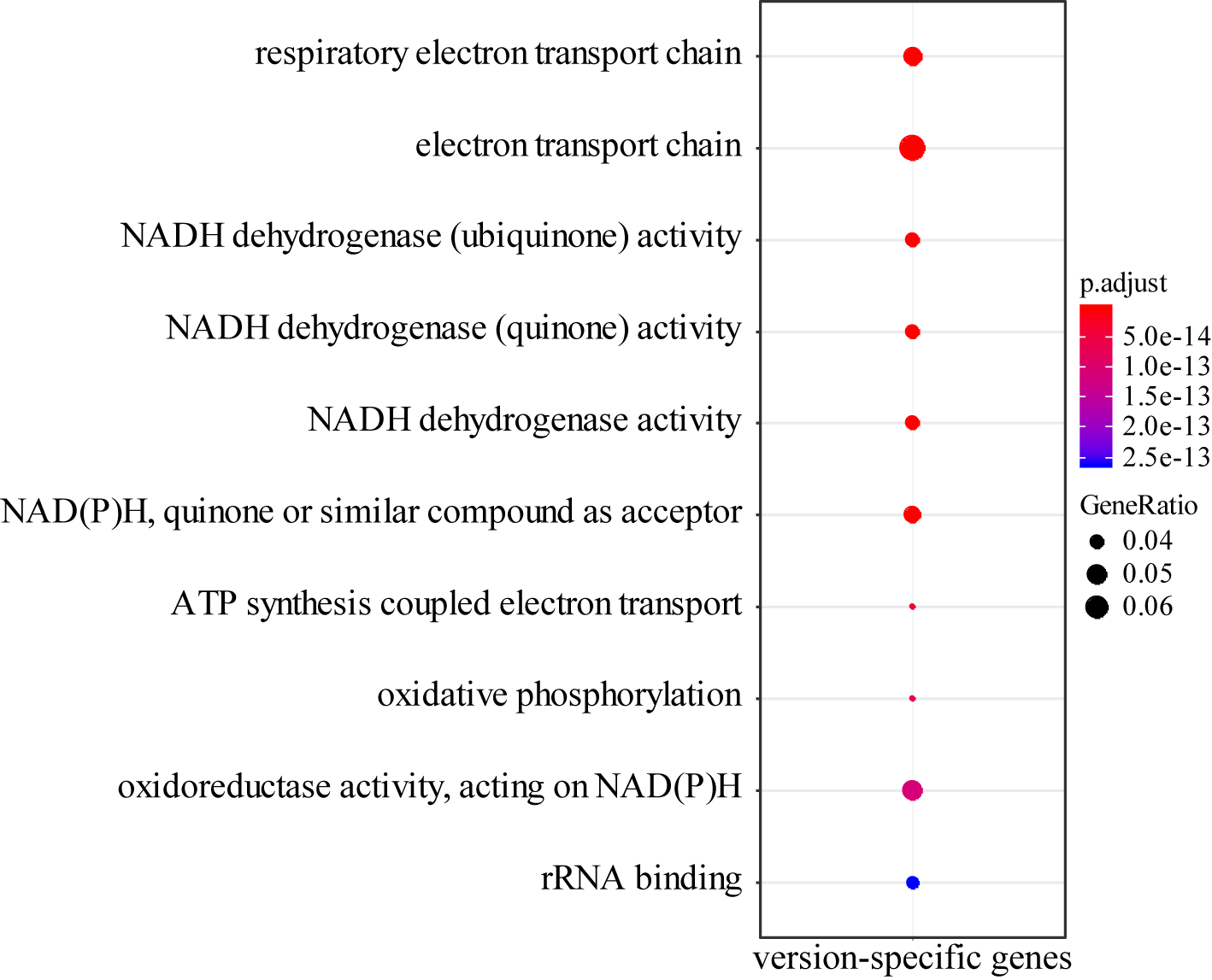


**Fig. S3** GO enrichment analysis of these specific genes in *S. suchowensis* genome v2.0


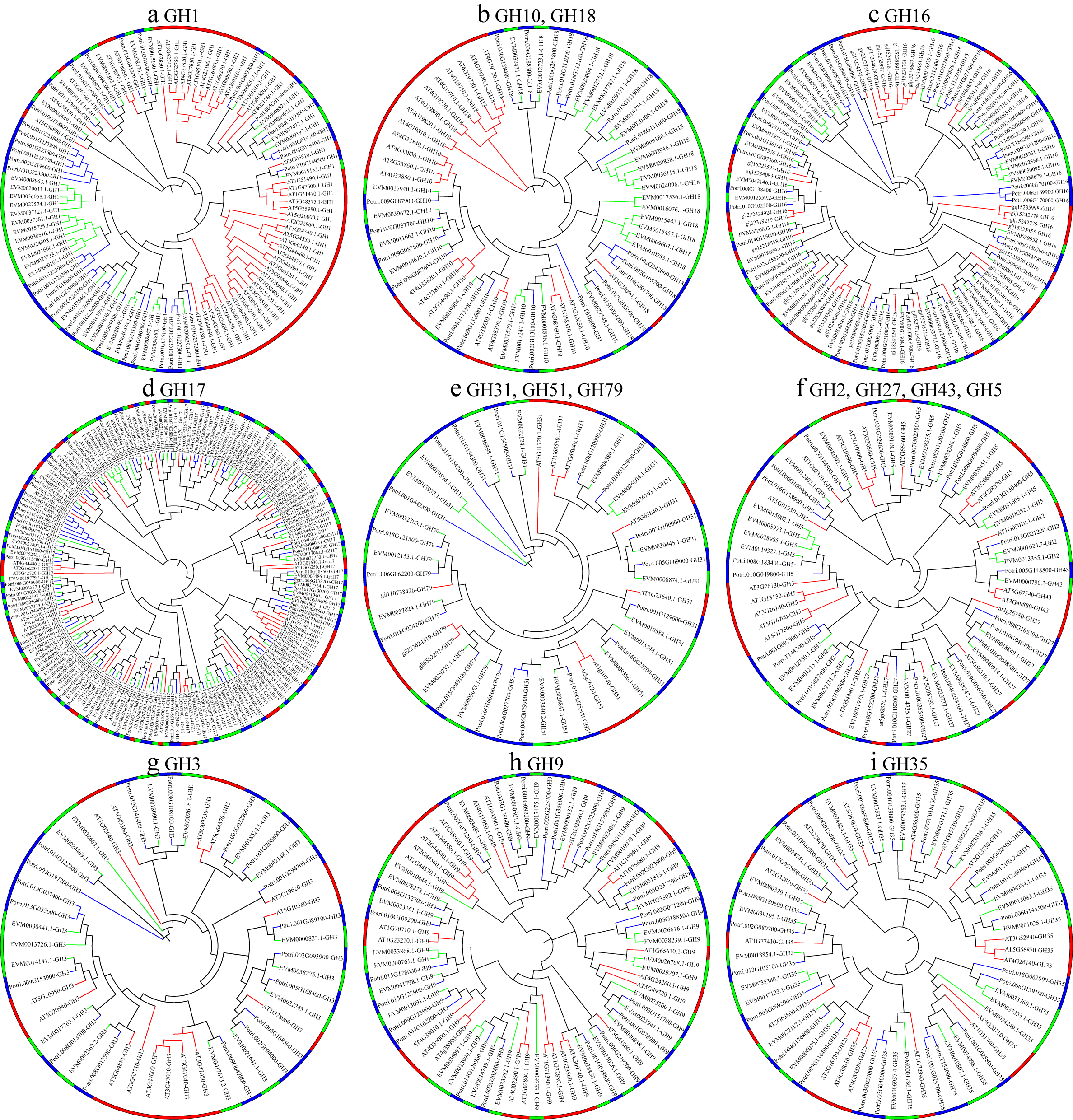


**Fig. S4** Phylogenetic analysis of glycoside hydrolase (GH) gene families in *Salix*, *Populus*, and *Arabidopsis*. Each protein is encoded with gene number and gene family name. Red, *A. thaliana* genome TAIR 11; Green, *S. suchowensis* genome v2.0; Blue, *P. trichocarpa* genome v3.0.


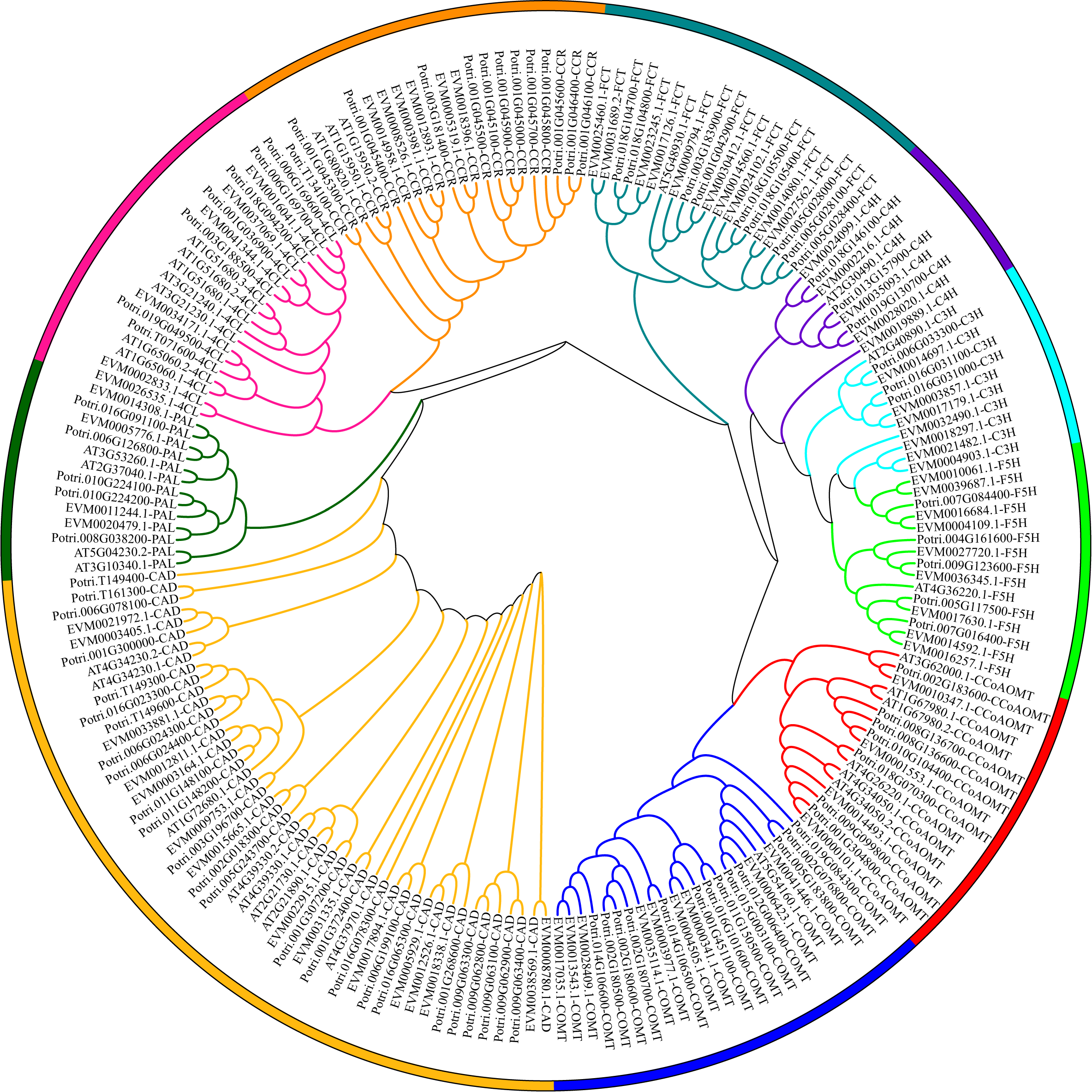


**Fig. S5** Phylogenetic analysis of gene families involved in lignin biosynthesis in *Salix*, *Populus*, and *Arabidopsis*. Each protein is encoded with gene number and gene family name. Different colors correspond to different clades.


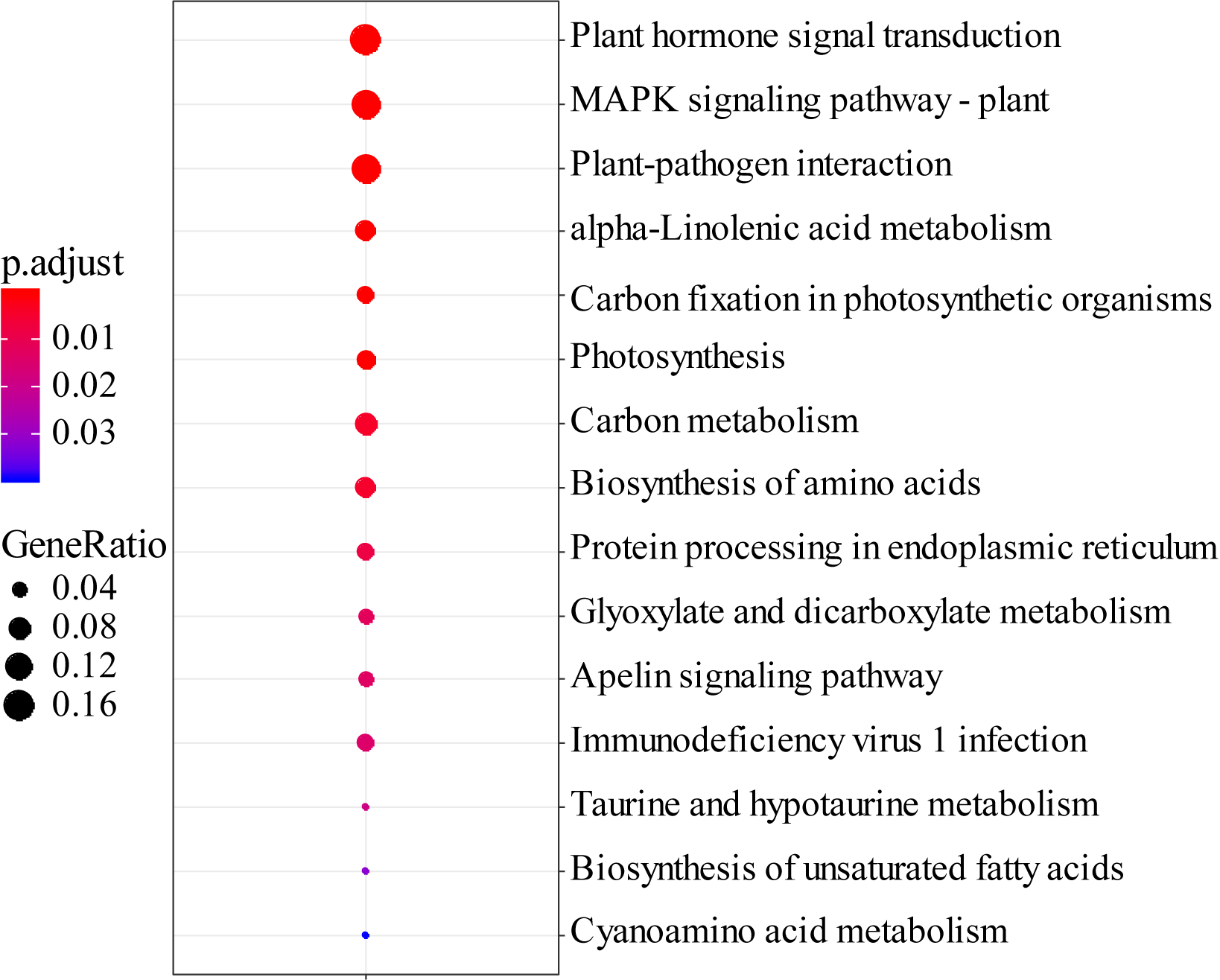


**Fig. S6** The KEGG pathway enrichment about genes involved in the salicylic acid biosynthesis and metabolism.
