# Supplementary material for: The chromosome-scale assembly of the willow genome provides insight into Salicaceae genome evolution
Source: Hortic Res. 2020 Apr 1;7:45. doi: 10.1038/s41438-020-0268-6 (PMC7109076; doi:10.1038/s41438-020-0268-6)
Supplement: Supplementary file 2 — Supplementary Tables [file 41438_2020_268_MOESM2_ESM.doc]

**Supplementary Tables**

Table S1. Statistics of PacBio sequencing data

| **Items** | **Polymerase reads** | **Subreads** |
| --- | --- | --- |
| Total number of reads | 2,578,121 | 4,170,347 |
| Total number of sequenced bases (bp) | 33,287,422,400 | 33,211,727,058 |
| Mean reads length (bp) | 12,964 | 7,981 |
| Max reads length (bp) | 18,880 | 12,965 |
| N50 (bp) | 16,197 | 10,767 |
| Coverage (X)* | 78.32 | 78.15 |
| * Coverage (X) = read length × read count / estimated genome size | | |

Table S2. Statistics of pre-assembly of *S. suchowensis* genome v2.0

| **Contig number** | **Contig length (bp)** | **Contig N50 (bp)** | **Contig max (bp)** | **GC content (%)** |
| --- | --- | --- | --- | --- |
| 3,444 | 356,983,684 | 263,908 | 2,191,170 | 34.94 |

Table S3. Statistics of Hi-C data and assessment

| **Statistics of Hi-C data** | | | |
| --- | --- | --- | --- |
| Number of read pairs | Number of bases (bp) | GC content (%) | % ≥ Q30 |
| 135,189,893 | 40,488,354,876 | 37.93 | 91.77 |
| **Statistics of mapping** | | | |
| Mapping type | | Number of reads | Ratio (%) |
| Total read pairs | | 135,189,893 | 100 |
| Mapped reads | | 250,256,420 | 92.56 |
| Unique mapped read pairs | | 72,466,875 | 53.6 |
| **Statistics of valid Hi-C data** | | | |
| Type | | Number of reads | Ratio (%) |
| Unique paired alignments | | 72,466,875 | 100 |
| Valid interaction pairs | | 59,036,645 | 81.47 |
| Dangling end pairs | | 4,794,040 | 6.62 |
| Re-ligation pairs | | 1,196,849 | 1.65 |
| Self-cycle pairs | | 3,740,789 | 5.16 |
| Dumped pairs | | 3,698,552 | 5.1 |

Supplemental Table 4. Summary of chromosome level assembly based on Hi-C data.

| **chromosome** | **Number of clustered contigs** | **Length of clustered contigs (bp)** | **Number of anchored and oriented contigs** | **Length of anchored and oriented contigs (bp)** | **Gap base (N)** | **N (%)** |
| --- | --- | --- | --- | --- | --- | --- |
| Chr_I | 324 | 37,766,479 | 266 | 34,977,238 | 26,500 | 0.08 |
| Chr_II | 157 | 20,174,127 | 128 | 19,155,828 | 12,700 | 0.07 |
| Chr_III | 124 | 16,424,621 | 90 | 15,296,090 | 8,900 | 0.06 |
| Chr_IV | 155 | 18,089,372 | 137 | 17,423,472 | 13,600 | 0.08 |
| Chr_V | 176 | 20,768,261 | 136 | 18,972,321 | 13,500 | 0.07 |
| Chr_VI | 153 | 21,586,240 | 124 | 20,430,144 | 12,300 | 0.06 |
| Chr_VII | 140 | 15,229,358 | 97 | 13,358,913 | 9,600 | 0.07 |
| Chr_VIII | 96 | 15,115,366 | 63 | 12,987,319 | 6,200 | 0.05 |
| Chr_IX | 93 | 11,936,783 | 72 | 11,223,490 | 7,100 | 0.06 |
| Chr_X | 95 | 17,458,125 | 75 | 16,851,236 | 7,400 | 0.04 |
| Chr_XI | 196 | 18,060,219 | 165 | 16,776,717 | 16,400 | 0.1 |
| Chr_XII | 138 | 13,343,648 | 87 | 10,670,725 | 8,600 | 0.08 |
| Chr_XIII | 156 | 16,220,857 | 129 | 14,960,644 | 12,800 | 0.09 |
| Chr_XIV | 109 | 14,131,296 | 81 | 12,877,745 | 8,000 | 0.06 |
| Chr_XV | 169 | 16,440,265 | 110 | 12,673,294 | 10,900 | 0.09 |
| Chr_XVI | 130 | 17,589,335 | 111 | 16,915,978 | 11,000 | 0.07 |
| Chr_XVII | 158 | 16,357,816 | 118 | 14,043,593 | 11,700 | 0.08 |
| Chr_XVIII | 128 | 14,186,142 | 113 | 13,419,317 | 11,200 | 0.08 |
| Chr_XIX | 216 | 18,794,577 | 160 | 16,863,940 | 15,900 | 0.09 |
| Total | 2,913 | 339,672,887 | 2,262 | 309,878,004 | 224,300 | 1.37 |

Table S5. Summary statistics of the annotated transposable elements in the *S. suchowensis* genome v2.0.

| **Repeat type** | **Number of elements** | **Length（bp）** | **Percentage in Genome (%)** |
| --- | --- | --- | --- |
| Class I: Retrotransposons | **244,639** | **100,777,199** | **28.27** |
| LTR-Retrotransposons | 167,895 | 77,843,034 | 21.84 |
| LTR/Copia | 74,224 | 37,552,569 | 10.53 |
| LTR/Gypsy | 89,118 | 38,724,555 | 10.86 |
| LTR-other | 4,553 | 1,565,910 | 0.44 |
| Non-LTR Retrotransposons | 24,605 | 6,667,747 | 1.87 |
| LINE | 19,709 | 5,913,236 | 1.66 |
| SINE | 4,896 | 754,511 | 0.21 |
| Other Retrotransposons | 52,139 | 16,266,418 | 4.56 |
| Class II: DNA Transposons | **68,515** | **18,964,446** | **5.32** |
| Academ | 1 | 34 | 0.00 |
| ISL2EU | 1 | 31 | 0.00 |
| Kolobok | 552 | 41,002 | 0.01 |
| MuDR | 2,435 | 247,027 | 0.07 |
| Novosib | 79 | 6,370 | 0.00 |
| Sola | 14 | 1,089 | 0.00 |
| Crypton | 16 | 880 | 0.00 |
| Helitron | 30,248 | 8,680,370 | 2.44 |
| MITE | 1,021 | 206,874 | 0.06 |
| Maverick | 891 | 354,029 | 0.10 |
| TIR | 30,430 | 8,827,724 | 2.48 |
| DNA-other | 2,827 | 599,016 | 0.17 |
| Potential Host Genes | **40,846** | **10,176,181** | **2.85** |
| SSR | **4,311** | **1,125,181** | **0.32** |
| Unknown | **133,010** | **37,459,884** | **10.51** |
| Total | 491,321 | 168,502,891 | 47.27 |

Table S6. Details of the protein-coding genes in pseudo-chromosomes of *S. suchowensis* genome v2.0

| **chromosome** | **Anchored annotated genes** | **length of anchored genes (bp)** | **Anchored and oriented annotated genes** | **length of anchored and oriented genes (bp)** |
| --- | --- | --- | --- | --- |
| Chr_I | 3,546 | 12,249,613 | 3,179 | 11,318,815 |
| Chr_II | 2,335 | 8,181,680 | 2,194 | 7,834,505 |
| Chr_III | 1,859 | 6,710,757 | 1,738 | 6,391,239 |
| Chr_IV | 1,842 | 6,516,713 | 1,769 | 6,348,516 |
| Chr_V | 2,250 | 7,729,271 | 2,082 | 7,299,774 |
| Chr_VI | 2,449 | 8,609,651 | 2,317 | 8,290,744 |
| Chr_VII | 1,416 | 4,859,886 | 1,281 | 4,486,563 |
| Chr_VIII | 1,858 | 6,359,014 | 1,600 | 5,540,770 |
| Chr_IX | 1,480 | 4,899,878 | 1,410 | 4,758,508 |
| Chr_X | 2,132 | 7,612,398 | 2,040 | 7,388,968 |
| Chr_XI | 1,484 | 5,362,771 | 1,357 | 5,080,625 |
| Chr_XII | 1,176 | 4,204,803 | 1,011 | 3,617,636 |
| Chr_XIII | 1,486 | 5,606,337 | 1,364 | 5,209,268 |
| Chr_XIV | 1,698 | 5,807,540 | 1,542 | 5,396,981 |
| Chr_XV | 1,501 | 5,501,175 | 1,266 | 4,736,035 |
| Chr_XVI | 2,058 | 7,480,769 | 1,972 | 7,283,069 |
| Chr_XVII | 1,418 | 5,162,907 | 1,234 | 4,583,237 |
| Chr_XVIII | 1,315 | 4,959,221 | 1,231 | 4,735,001 |
| Chr_XIX | 1,393 | 5,158,899 | 1,276 | 4,795,144 |
| Total  ( % of genome ) | 34,696  (93.93%) | 122,973,283  (34.45%) | 31,863  (86.26%) | 115,095,398  (32.24%) |

Table S7. Mapping summary of RNA-Seq data to protein-coding genes in *S. suchowensis* genome v2.0

| **Samples** | **Expressed genes** | **Unexpressed genes** | **Genome coverage** |
| --- | --- | --- | --- |
| Root | 28,615 | 3,248 | 89.81% |
| Stem | 27,985 | 3,878 | 87.83% |
| Leaf | 26,644 | 5,219 | 83.62% |
| Bud | 28,188 | 3,675 | 88.47% |
| Bark | 27,629 | 4,234 | 86.71% |

Table S8. Functional annotation of predicted protein-coding genes in the *S. suchowensis* genome v2.0

| **Database** | **Number of genes annotated** | **Percentage (%)** |
| --- | --- | --- |
| GO | 22,878 | 61.94 |
| KEGG | 13,224 | 35.80 |
| KOG | 19,524 | 52.86 |
| TrEMBL | 34,748 | 94.07 |
| NR | 36,135 | 97.83 |
| Total | 36,150 | 97.87 |

Table S9. Summary of non-coding RNA in the *S. suchowensis* genome v2.0

| **Type** | **Number** | **Average length (bp)** | **Total length (bp)** | **% of genome** |
| --- | --- | --- | --- | --- |
| miRNA | 172 | 116 | 20,013 | 0.004843 |
| rRNA | 261 | 963 | 251,420 | 0.060843 |
| tRNA | 846 | 74 | 62,958 | 0.015236 |

Table S10. The genomic comparison of *S. suchowensis* v1.0, *S. suchowensis* v2.0, *P. trichocarpa* v1.0, and *P. trichocarpa* v3.0

| **Type** | ***S. suchowensis* v2.0** | ***S. suchowensis* v1.0** | ***P. trichocarpa* v1.0** | ***P. trichocarpa* v3.0** |
| --- | --- | --- | --- | --- |
| Assembly size (Mb) | 356.5 | 303.8 | 410 | 434 |
| Assembly size of chromosomes (Mb) | 310 | 229 | 385 | 394.5 |
| Number of protein-coding genes | 36,937 | 26,599 | 45,555 | 41,335 |
| Number of genes on chromosomes  ( % of genome ) | 31,863  (86.3%) | 24,931  (93.7%) | 30,260  (66.4%) | 39,514  (95.6%) |
| Number of complete BUSCOs (%) | 1336 (94.8%) | 1290 (89.6%) | 1306 (90.7%) | 1406 (97.6%) |
| Number of complete and single-copy BUSCOs (%) | 1095 (76.0%) | 1169 (81.2%) | 1112 (77.2%) | 1158 (80.4%) |
| Number of complete and duplicated BUSCOs (%) | 271 (18.8%) | 121 (8.4%) | 194 (13.5%) | 248 (17.2%) |

Table S11. The alignment results of BAC sequences against the *S. suchowensis* genome v2.0 and v1.0

|  | | | ***S. suchowensis* v2.0** | | ***S. suchowensis* v1.0** | |
| --- | --- | --- | --- | --- | --- | --- |
| **BAC ID** | **BAC length (bp)** | **Chromosome** | **Total length of mapped sequences** | **Coverage (%)** | **Total length of mapped sequences** | **Coverage (%)** |
| BAC_1 | 124,375 | chr15 | 124,025 | 99.7 | 119,080 | 95.7 |
| BAC_2 | 100,141 | chr15 | 99,221 | 99.1 | 94,372 | 94.2 |
| BAC_3 | 185,279 | chr16 | 183,557 | 99.1 | 168,172 | 90.8 |
| BAC_4 | 161,698 | chr01 | 157,435 | 97.4 | 136,759 | 84.6 |
| BAC_5 | 96,086 | chr02 | 96,086 | 100.0 | 93,047 | 96.8 |
| BAC_6 | 100,882 | chr02 | 100,299 | 99.4 | 98,123 | 97.3 |
| BAC_7 | 123,265 | chr14 | 123,181 | 99.9 | 113,611 | 92.2 |
| Total | 891,726 | -- | 883,804 | 99.2 | 823,164 | 93.1 |

Table S12. Summary of DNA short reads mapping in the *S. suchowensis* genome v1.0 and v2.0.

| **Version** | **ID** | **Total reads** | **Data (Gb)** | **Mapped reads** | **Mapped reads ratio** | **Mapped pairs** | **Mapped pairs ratio** |
| --- | --- | --- | --- | --- | --- | --- | --- |
|  |  |  |  |  |  |  |  |
| *S. suchowensis* v2.0 | L1_LIB | 36,323,166 | 4.01 | 36,137,610 | 99.49% | 34,668,728 | **95.99%** |
|  | L2_LIB | 36,428,031 | 4.01 | 36,244,849 | 99.50% | 35,282,790 | **97.28%** |
|  | L3_LIB | 36,617,892 | 4.18 | 36,493,346 | 99.66% | 35,712,112 | **97.97%** |
|  | L5_LIB | 36,954,879 | 4.01 | 36,800,010 | 99.58% | 31,518,958 | **85.64%** |
|  | L6_LIB | 37,193,896 | 4.01 | 37,025,504 | 99.55% | 29,989,882 | **80.95%** |
|  | L7_LIB | 36,871,069 | 4.01 | 36,711,925 | 99.57% | 30,522,856 | **83.10%** |
|  | L8_LIB | 36,710,132 | 4.01 | 36,554,434 | 99.58% | 33,595,582 | **91.70%** |
| *S. suchowensis* v1.0 | L1_LIB | 38,988,579 | 4.01 | 38,948,870 | 99.90% | 35,036,472 | **91.19%** |
|  | L2_LIB | 39,050,394 | 4.01 | 39,011,688 | 99.90% | 36,022,556 | **93.46%** |
|  | L3_LIB | 39,388,158 | 4.18 | 39,359,521 | 99.93% | 36,829,886 | **95.22%** |
|  | L5_LIB | 39,565,223 | 4.01 | 39,535,713 | 99.93% | 26,573,674 | **68.03%** |
|  | L6_LIB | 39,592,607 | 4.01 | 39,561,330 | 99.92% | 24,707,362 | **63.26%** |
|  | L7_LIB | 39,489,886 | 4.01 | 39,459,316 | 99.92% | 25,277,540 | **64.77%** |
|  | L8_LIB | 39,332,755 | 4.01 | 39,305,711 | 99.93% | 27,615,338 | **70.82%** |

Table S13. Evaluation of the genome assembly with the RNA-Seq data

| **Version** | **Unigene** | **Number** | **Percent** | **with >50% sequence in one scaffold** | | **with >90% sequence in one scaffold** | | |
| --- | --- | --- | --- | --- | --- | --- | --- | --- |
|  |  |  |  | **Number** | **Percent** | | **Number** | **Percent** |
| *S. suchowensis* v2.0 | All | 85,962 | 99.31% | 85,136 | 99.04% | | 83,331 | 96.94% |
|  | >=500 bp | 59,354 | 99.40% | 58,797 | 99.06% | | 57,996 | 97.71% |
|  | >=1000 bp | 34,542 | 99.45% | 34,212 | 99.04% | | 33,837 | 97.96% |
| *S. suchowensis* v1.0 | All | 85,962 | 97.60% | 84,902 | 98.80% | | 74,786 | 87.00% |
|  | >=500 bp | 57,805 | 97.70% | 57,292 | 99.10% | | 48,152 | 83.30% |
|  | >=1000 bp | 42,371 | 97.60% | 42,028 | 99.20% | | 34,393 | 81.20% |

Table S14. Details of the specific protein-coding genes in *S. suchowensis* genome v2.0

| **Chromosome** | **Number of specific genes in the unordered chromosome** | **Number of specific genes in the anchored and oriented chromosome** | **Total** |
| --- | --- | --- | --- |
| Chr_I | 188 | 267 | 455 |
| Chr_II | 49 | 140 | 189 |
| Chr_III | 49 | 111 | 160 |
| Chr_IV | 38 | 134 | 172 |
| Chr_V | 60 | 141 | 201 |
| Chr_VI | 71 | 161 | 232 |
| Chr_VII | 55 | 112 | 167 |
| Chr_VIII | 45 | 106 | 151 |
| Chr_IX | 34 | 80 | 114 |
| Chr_X | 28 | 143 | 171 |
| Chr_XI | 56 | 123 | 179 |
| Chr_XII | 37 | 77 | 114 |
| Chr_XIII | 25 | 116 | 141 |
| Chr_XIV | 55 | 86 | 141 |
| Chr_XV | 70 | 100 | 170 |
| Chr_XVI | 34 | 146 | 180 |
| Chr_XVII | 61 | 121 | 182 |
| Chr_XVIII | 38 | 123 | 161 |
| Chr_XIX | 65 | 183 | 248 |
| Unclustered | -- | -- | 1,754 |

Table S15. Gene families related to wood formation in *Salix*, *Populus*, and *Arabidopsis*

| **Classify** | **Family** | **Gene Name** | ***A. thaliana*** | ***P. trichocarpa*** | ***S. suchowensis*** |
| --- | --- | --- | --- | --- | --- |
| Cellulose and hemicellulose | FUT | fucosyltransferase | 11 | 10 | 10 |
|  | RGP | hydroxyproline-rich glycoprotei | 4 | 7 | 5 |
|  | XXT | xyloglucan xylosyltransferases | 4 | 5 | 6 |
|  | GH1 | glycoside hydrolase 1 | 48 | 32 | 32 |
|  | GH10 | glycoside hydrolase 10 | 12 | 7 | 8 |
|  | GH16 | glycoside hydrolase 16 | 33 | 42 | 35 |
|  | GH17 | glycoside hydrolase 17 | 51 | 71 | 68 |
|  | GH18 | glycoside hydrolase 18 | 10 | 12 | 20 |
|  | GH2 | glycoside hydrolase 2 | 2 | 3 | 4 |
|  | GH27 | glycoside hydrolase 27 | 4 | 8 | 6 |
|  | GH3 | glycoside hydrolase 3 | 15 | 18 | 16 |
|  | GH31 | glycoside hydrolase 31 | 5 | 9 | 10 |
|  | GH35 | glycoside hydrolase 35 | 17 | 23 | 24 |
|  | GH43 | glycoside hydrolase 43 | 2 | 1 | 1 |
|  | GH5 | glycoside hydrolase 5 | 13 | 13 | 13 |
|  | GH51 | glycoside hydrolase 51 | 2 | 4 | 4 |
|  | GH79 | glycoside hydrolase 79 | 3 | 5 | 5 |
|  | GH9 | glycoside hydrolase 9 | 25 | 24 | 29 |
| Lignin | 4CL | 4-coumarate: CoA ligase | 7 | 7 | 6 |
|  | C3H | p-coumarate-3-hydroxylase | 1 | 3 | 7 |
|  | C4H | Trans-cinnamate 4-hydroxylase | 1 | 3 | 5 |
|  | CAD | cinnamyl alcohol dehydrogenase | 8 | 25 | 15 |
|  | CCoAOMT | Caffeoyl coenzyme A-3-O-methyltransferase | 7 | 7 | 4 |
|  | CCR | cinnamoyl CoA reductase | 3 | 13 | 6 |
|  | COMT | caffeie acid O-methyltransferase | 1 | 13 | 9 |
|  | F5H | ferulate-5-hydroxylase | 1 | 5 | 9 |
|  | HCT | hydroxycinnamoyl-CoA shikimate/quinate hydroxycinnamoyl transferase | 1 | 9 | 10 |
|  | PAL | phenylalanine ammonia-lyase | 4 | 5 | 4 |

Table S16. Statistics of Ka / Ks values in the salicoid duplicates in the *S. suchowensis* and *P. trichocarpa* chromosomes

| Chromosome | salicoid duplicates in *S. suchowensis* | | | salicoid duplicates in *P. trichocarpa* | | |
| --- | --- | --- | --- | --- | --- | --- |
|  | No. of genes | Range of Ka/Ks values | Mean of Ka/Ks | No. of genes | Range of Ka/Ks values | Mean of Ka/Ks |
| Chr_I | 1,262 | 0 - 0.98 | 0.28 | 2,170 | 0 - 1 | 0.31 |
| Chr_II | 1,020 | 0 - 0.99 | 0.28 | 1,460 | 0 - 0.96 | 0.31 |
| Chr_III | 908 | 0 - 0.92 | 0.28 | 1,180 | 0 - 1 | 0.31 |
| Chr_IV | 847 | 0 - 0.97 | 0.27 | 1,111 | 0 - 1 | 0.3 |
| Chr_V | 1,027 | 0 - 0.99 | 0.29 | 1,348 | 0 - 0.97 | 0.3 |
| Chr_VI | 1,009 | 0 - 0.87 | 0.27 | 1,361 | 0 - 0.99 | 0.31 |
| Chr_VII | 485 | 0 - 0.86 | 0.28 | 670 | 0 - 0.97 | 0.3 |
| Chr_VIII | 911 | 0 - 0.93 | 0.29 | 1,367 | 0 - 0.96 | 0.31 |
| Chr_IX | 678 | 0 - 0.99 | 0.27 | 905 | 0 - 0.97 | 0.3 |
| Chr_X | 897 | 0 - 0.84 | 0.28 | 1,371 | 0 - 0.96 | 0.31 |
| Chr_XI | 565 | 0 - 0.97 | 0.29 | 760 | 0 - 1 | 0.31 |
| Chr_XII | 533 | 0 - 0.92 | 0.29 | 733 | 0 - 0.92 | 0.32 |
| Chr_XIII | 513 | 0 - 0.84 | 0.29 | 647 | 0 - 0.97 | 0.31 |
| Chr_XIV | 552 | 0 - 0.9 | 0.3 | 875 | 0 - 0.91 | 0.32 |
| Chr_XV | 513 | 0 - 0.92 | 0.29 | 673 | 0 - 0.92 | 0.31 |
| Chr_XVI | 936 | 0 - 0.92 | 0.29 | 701 | 0 - 0.99 | 0.3 |
| Chr_XVII | 426 | 0 - 0.91 | 0.3 | 576 | 0 - 0.99 | 0.32 |
| Chr_XVIII | 483 | 0 - 0.98 | 0.29 | 671 | 0 - 0.98 | 0.32 |
| Chr_XIX | 483 | 0 - 0.96 | 0.3 | 487 | 0 - 0.98 | 0.34 |
